# Bis(thio­cyanato-κ*N*)[tris­(2-pyridylmeth­yl)amine-κ^4^ *N*,*N*′,*N*′′,*N*′′′]nickel(II) methanol hemisolvate

**DOI:** 10.1107/S1600536809021722

**Published:** 2009-06-13

**Authors:** Sajini R. Randeniya, Richard E. Norman

**Affiliations:** a Department of Chemistry, Box 2117, Sam Houston State University, Huntsville, TX 77341, USA

## Abstract

The title complex, [Ni(NCS)_2_(C_18_H_18_N_4_)]·0.5CH_3_OH, consists of two crystallographically distinct complexes and a methanol solvent mol­ecule. The Ni^II^ complexes are pseudo-octa­hedral six-coordinate, with the tris­(2-pyridylmeth­yl)amine (TPA) ligand providing four N atoms and two N-bound thio­cyanates providing the final two N atoms. The distances and angles are typical for Ni^II^–TPA complexes. The compound has unit-cell parameters that are surprisingly similar to the previously reported hydrate.

## Related literature

For the synthesis and characterization (including a structural determination) of the analogous hydrate, see: Yan *et al.* (1999 ▶); Xu *et al.* (2003 ▶). For a description of the Cambridge Crystallographic Database, see: Allen (2002 ▶). For related structures, see: Tong *et al.* (1999 ▶, 2000 ▶); Orpen *et al.* (1989 ▶); Nagataki *et al.* (2006 ▶).
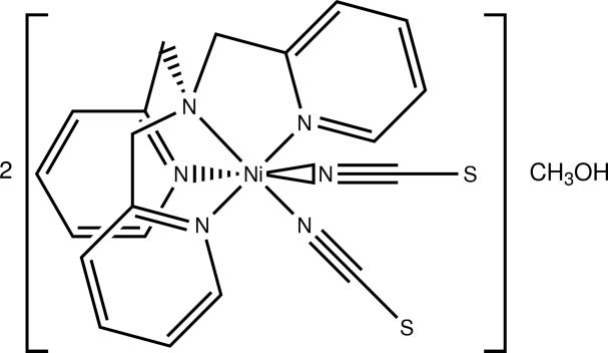


## Experimental

### 

#### Crystal data

[Ni(NCS)_2_(C_18_H_18_N_4_)]·0.5CH_4_O
*M* *_r_* = 481.26
Triclinic, 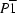

*a* = 9.482 (1) Å
*b* = 15.523 (3) Å
*c* = 15.839 (3) Å
α = 73.415 (9)°
β = 87.517 (12)°
γ = 76.205 (10)°
*V* = 2169.2 (7) Å^3^
*Z* = 4
Mo *K*α radiation
μ = 1.11 mm^−1^
*T* = 90 K
0.20 × 0.07 × 0.04 mm

#### Data collection

Nonius KappaCCD diffractometer
Absorption correction: multi-scan (*SCALEPACK*; Otwinowski & Minor, 1997 ▶) *T*_min_ = 0.799, *T*_max_ = 0.957
16372 measured reflections
8526 independent reflections
5290 reflections with *I* > 2σ(*I*)
*R*_int_ = 0.049

#### Refinement

*R*[*F* ^2^ > 2σ(*F* ^2^)] = 0.075
*wR*(*F* ^2^) = 0.137
*S* = 0.91
5290 reflections
541 parameters
H-atom parameters constrained
Δρ_max_ = 1.30 e Å^−3^
Δρ_min_ = −0.65 e Å^−3^

### 

Data collection: *COLLECT* (Nonius, 2000 ▶); cell refinement: *SCALEPACK* (Otwinowski & Minor, 1997 ▶); data reduction: *DENZO* (Otwinowski & Minor, 1997 ▶) and *SCALEPACK*; program(s) used to solve structure: *SIR92* (Altomare *et al.*, 1993 ▶); program(s) used to refine structure: *TEXSAN for Windows* (Molecular Structure Corporation, 1999 ▶); molecular graphics: *ORTEPII* (Johnson, 1976 ▶); software used to prepare material for publication: *TEXSAN for Windows*.

## Supplementary Material

Crystal structure: contains datablocks global, I. DOI: 10.1107/S1600536809021722/nc2147sup1.cif

Structure factors: contains datablocks I. DOI: 10.1107/S1600536809021722/nc2147Isup2.hkl

Additional supplementary materials:  crystallographic information; 3D view; checkCIF report

## Figures and Tables

**Table 1:** Selected geometric parameters (Å, °)

Ni1—N11	2.079 (4)
Ni1—N12	2.086 (4)
Ni1—N13	2.093 (4)
Ni1—N14	2.122 (4)
Ni1—N122	2.073 (4)
Ni1—N144	2.040 (5)
Ni2—N21	2.049 (5)
Ni2—N22	2.079 (4)
Ni2—N23	2.061 (5)
Ni2—N24	2.126 (4)
Ni2—N222	2.102 (5)
Ni2—N244	2.043 (5)

**Fig. 1.**
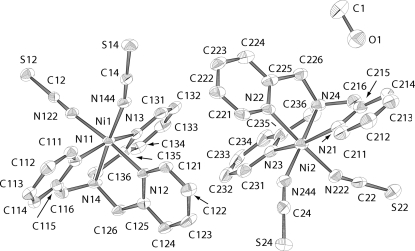
Perspective drawing of the asymmetric unit with displacement ellipsoids drawn at the 50% probability level and hydrogen atoms removed.

## supplementary crystallographic information

### Comment

2 [Ni(TPA)(NCS)_2_]^.^H_2_O was reported previously (Yan, *et al.*, 1999 and Xu, *et al.*, 2003). The Cambridge Crystallographic Data Centre codes for the compound are VILQOW and VILQOW01, Respectively (Allen, 2002). The unit-cell parameters for the hydrate and the methanol solvate are surprisingly similar.

In the latter paper (Xu, *et al.*, 2003) the water of hydration is not mentioned in the discussion of the structure other than an indication of its presence. Figure 1 of that report shows O1 bound to O1', but no details about these oxygen atoms are given. In the CIF for VILQOW, O1' is not listed and O1 corresponds quite well to our O1 and exhibits the same pattern of contacts.

In the current report, there are two crystallographically distinct Ni^II^ complexes and one methanol in the asymmetric unit. The Ni complexes are pseudo-octahedral, 6 coordinate, with TPA providing 4 nitrogen atoms, and the N-bound thiocyanates provide 2 additional nitrogen atoms. The longest nickel-nitrogen bond in each complex involves the tertiary amine of TPA, and the shortest nickel-nitrogen bond in each complex involves the thiocyanate *trans* to the tertiary amine, a pattern seen previously in di-µ-halo-bis{[tris(2-pyridylmethyl)- amine-κ^4^*N*]nickel(II)} bis(triethylammonium) tetraperchlorate where halo is chloro and bromo (Tong, *et al.*, 1999 & Tong, *et al.*, 2000) where the longest nickel-nitrogen bond involves the tertiary amine of TPA and the shortest nickel-halo bond is *trans* to the tertiary amine. The average nickel-pyridyl nitrogen distance (2.074 Å) falls in the lower quartile for similar compounds (2.096 Å, Orpen, *et al.*, 1989). The average nickel-isothiocyanate nitrogen distance (2.064 Å) is the median for similar compounds (Orpen, *et al.*, 1989).

The methanol hydrogen bonds through H1 to the sulfur atom of an adjacent thiocyanate (S22, 1 - *x*, 1 - *y*, -*z*).

### Experimental

Tris[(2-pyridinium)methyl]amine perchlorate (0.1954 g, 0.330 mmol) and triethylamine (0.105 ml, 0.75 mmol) were dissolved in 20 ml MeOH. Ni(ClO_4_)_2_^.^6H_2_O (0.1879 g, 0.501 mmol) and NH_4_SCN (0.076 g, 1.0 mmol) were dissolved in 3 ml MeOH and this solution was added to the ligand solution and stirred for 30 minutes. A powdery purple precipitate formed immediately upon mixing the two solutions. The mixture was gravity filtered. The filtrate was allowed to stand undisturbed for two days, and small purple crystals formed.

### Refinement

With the exception of the hydrogen atom bound to the methanol oxygen, which was located in a difference map, the hydrogen atoms were placed in calculated positions and refined using a riding model. The position parameters of the hydrogen atom bound to the methanol oxygen were not refined. All hydrogen atoms were assigned thermal parameters 1.2 times larger than the atoms to which they are bound.

### Crystal data

**Table:**

[Ni(NCS)_2_(C_18_H_18_N_4_)]·0.5CH_4_O	*Z* = 4
*M**_r_* = 481.26	*F*(000) = 996.00
Triclinic, *P*1	*D*_x_ = 1.473 Mg m^−^^3^
Hall symbol: -P 1	Mo *K*α radiation, λ = 0.7107 Å
*a* = 9.482 (1) Å	Cell parameters from 8223 reflections
*b* = 15.523 (3) Å	θ = 2.5–26.0°
*c* = 15.839 (3) Å	µ = 1.11 mm^−^^1^
α = 73.415 (9)°	*T* = 90 K
β = 87.517 (12)°	Needle, purple
γ = 76.205 (10)°	0.20 × 0.07 × 0.04 mm
*V* = 2169.2 (7) Å^3^	

### Data collection

**Table:**

Nonius KappaCCD with an Oxford Cryosystems Cryostream cooler diffractometer	8526 independent reflections
Radiation source: fine-focus sealed tube	5290 reflections with *I* > 2σ(*I*)
graphite	*R*_int_ = 0.049
ω and φ scans	θ_max_ = 26.1°, θ_min_ = 2.6°
Absorption correction: multi-scan (*SCALEPACK*; Otwinowski & Minor, 1997)	*h* = −11→11
*T*_min_ = 0.799, *T*_max_ = 0.957	*k* = −19→19
16372 measured reflections	*l* = −19→19

### Refinement

**Table:**

Refinement on *F*^2^	0 restraints
Least-squares matrix: full	0 constraints
*R*[*F*^2^ > 2σ(*F*^2^)] = 0.075	H-atom parameters constrained
*wR*(*F*^2^) = 0.137	Weighting scheme based on measured s.u.'s *w* = 1/[σ^2^(*F*_o_) + 0.0025|*F*_o_|^2^]
*S* = 0.91	(Δ/σ)_max_ = 0.0002
5290 reflections	Δρ_max_ = 1.30 e Å^−^^3^
541 parameters	Δρ_min_ = −0.65 e Å^−^^3^

### Special details

**Table:**

Refinement. Refinement of *F*^2^. The weighted *R*-factor *wR* and goodness of fit are based on *F*^2^, conventional *R*-factors *R* are based on *F. R*-factors based on *F*^2^ are statistically about twice as large as those based on *F*.

### Fractional atomic coordinates and isotropic or equivalent isotropic displacement parameters (Å^2^)

**Table:**

	*x*	*y*	*z*	*U*_iso_*/*U*_eq_	
Ni1	0.17625 (7)	0.08947 (4)	0.73918 (4)	0.0246 (2)	
Ni2	0.19950 (7)	0.38729 (5)	0.24438 (4)	0.0312 (2)	
S12	0.5106 (1)	−0.14619 (9)	0.95779 (9)	0.0330 (4)	
S14	0.2342 (2)	−0.13602 (10)	0.57919 (10)	0.0381 (4)	
S22	0.3107 (2)	0.67884 (10)	0.09424 (11)	0.0433 (5)	
S24	−0.2789 (2)	0.54238 (11)	0.28929 (11)	0.0425 (4)	
O1	0.7669 (5)	0.2924 (3)	0.1191 (3)	0.058 (1)	
N11	−0.0089 (4)	0.0566 (3)	0.8022 (3)	0.0277 (12)	
N12	0.0414 (4)	0.2034 (3)	0.6520 (3)	0.0241 (11)	
N13	0.3559 (4)	0.1477 (3)	0.7061 (3)	0.0254 (11)	
N14	0.1426 (4)	0.1810 (3)	0.8192 (3)	0.0266 (11)	
N21	0.1672 (5)	0.3851 (3)	0.1180 (3)	0.0344 (13)	
N22	0.2247 (5)	0.2452 (3)	0.2949 (3)	0.0310 (12)	
N23	0.2990 (5)	0.3833 (3)	0.3590 (3)	0.0348 (13)	
N24	0.4193 (5)	0.3489 (3)	0.2087 (3)	0.0323 (13)	
N122	0.3083 (5)	−0.0145 (3)	0.8355 (3)	0.0294 (12)	
N144	0.1977 (4)	0.0090 (3)	0.6555 (3)	0.0291 (12)	
N222	0.2045 (5)	0.5275 (3)	0.1969 (3)	0.0354 (13)	
N244	−0.0113 (5)	0.4219 (3)	0.2804 (3)	0.037 (1)	
C1	0.8645 (7)	0.2053 (5)	0.1528 (5)	0.058 (2)	
C12	0.3932 (5)	−0.0685 (3)	0.8853 (3)	0.0232 (13)	
C14	0.2114 (5)	−0.0509 (4)	0.6239 (3)	0.027 (1)	
C22	0.2493 (6)	0.5897 (4)	0.1551 (4)	0.034 (2)	
C24	−0.1227 (6)	0.4713 (4)	0.2846 (3)	0.032 (2)	
C111	−0.0950 (6)	0.0084 (4)	0.7802 (4)	0.032 (2)	
C112	−0.2169 (6)	−0.0074 (4)	0.8272 (4)	0.037 (2)	
C113	−0.2548 (6)	0.0287 (4)	0.8975 (4)	0.037 (2)	
C114	−0.1669 (6)	0.0797 (4)	0.9192 (4)	0.032 (2)	
C115	−0.0444 (6)	0.0910 (4)	0.8713 (3)	0.029 (1)	
C116	0.0631 (6)	0.1389 (4)	0.8967 (3)	0.032 (2)	
C121	−0.0096 (5)	0.2078 (3)	0.5723 (3)	0.028 (1)	
C122	−0.0967 (6)	0.2869 (4)	0.5180 (4)	0.036 (2)	
C123	−0.1332 (6)	0.3652 (4)	0.5473 (4)	0.039 (2)	
C124	−0.0851 (6)	0.3615 (4)	0.6287 (4)	0.035 (2)	
C125	0.0030 (5)	0.2796 (3)	0.6796 (3)	0.026 (1)	
C126	0.0610 (6)	0.2749 (4)	0.7681 (3)	0.031 (2)	
C131	0.4451 (6)	0.1444 (4)	0.6387 (4)	0.032 (2)	
C132	0.5700 (6)	0.1761 (4)	0.6300 (4)	0.041 (2)	
C133	0.6032 (6)	0.2160 (4)	0.6923 (4)	0.038 (2)	
C134	0.5099 (6)	0.2217 (4)	0.7610 (4)	0.037 (2)	
C135	0.3879 (5)	0.1862 (3)	0.7663 (4)	0.028 (1)	
C136	0.2900 (5)	0.1834 (4)	0.8442 (4)	0.031 (2)	
C211	0.0431 (6)	0.3824 (4)	0.0825 (4)	0.040 (2)	
C212	0.0312 (7)	0.3855 (4)	−0.0050 (4)	0.046 (2)	
C213	0.1500 (7)	0.3892 (4)	−0.0562 (4)	0.042 (2)	
C214	0.2798 (6)	0.3924 (4)	−0.0211 (4)	0.040 (2)	
C215	0.2855 (6)	0.3884 (4)	0.0671 (4)	0.034 (2)	
C216	0.4216 (6)	0.3914 (4)	0.1122 (4)	0.037 (2)	
C221	0.1282 (6)	0.2013 (4)	0.3430 (4)	0.033 (2)	
C222	0.1646 (6)	0.1105 (4)	0.3901 (4)	0.038 (2)	
C223	0.3051 (6)	0.0609 (4)	0.3889 (4)	0.041 (2)	
C224	0.4054 (6)	0.1041 (4)	0.3368 (4)	0.040 (2)	
C225	0.3625 (6)	0.1967 (4)	0.2905 (4)	0.033 (2)	
C226	0.4634 (6)	0.2453 (4)	0.2305 (4)	0.035 (2)	
C231	0.2346 (7)	0.3841 (4)	0.4367 (4)	0.041 (2)	
C232	0.3106 (8)	0.3867 (4)	0.5092 (4)	0.047 (2)	
C233	0.4550 (9)	0.3892 (4)	0.4987 (4)	0.057 (2)	
C234	0.5236 (7)	0.3845 (4)	0.4202 (4)	0.044 (2)	
C235	0.4417 (6)	0.3819 (4)	0.3505 (4)	0.036 (2)	
C236	0.5053 (6)	0.3845 (4)	0.2609 (4)	0.041 (2)	
H1	0.7480	0.2917	0.0576	0.070*	
H11	0.8769	0.1927	0.2147	0.070*	
H12	0.8266	0.1585	0.1406	0.070*	
H13	0.9557	0.2062	0.1257	0.070*	
H111	−0.0714	−0.0153	0.7310	0.039*	
H112	−0.2745	−0.0429	0.8112	0.044*	
H113	−0.3388	0.0190	0.9302	0.044*	
H114	−0.1910	0.1064	0.9665	0.039*	
H115	0.1311	0.0947	0.9392	0.038*	
H116	0.0116	0.1860	0.9213	0.038*	
H121	0.0158	0.1538	0.5526	0.033*	
H122	−0.1307	0.2876	0.4622	0.043*	
H123	−0.1913	0.4212	0.5110	0.046*	
H124	−0.1116	0.4142	0.6503	0.042*	
H125	−0.0186	0.2949	0.8020	0.037*	
H126	0.1244	0.3156	0.7591	0.037*	
H131	0.4210	0.1191	0.5949	0.039*	
H132	0.6324	0.1708	0.5823	0.049*	
H133	0.6884	0.2390	0.6878	0.045*	
H134	0.5292	0.2495	0.8041	0.044*	
H135	0.3288	0.1297	0.8909	0.037*	
H136	0.2843	0.2370	0.8631	0.037*	
H211	−0.0391	0.3783	0.1185	0.048*	
H212	−0.0586	0.3851	−0.0291	0.055*	
H213	0.1442	0.3896	−0.1160	0.051*	
H214	0.3624	0.3972	−0.0567	0.048*	
H215	0.4269	0.4540	0.1011	0.045*	
H216	0.5044	0.3585	0.0891	0.045*	
H221	0.0308	0.2352	0.3441	0.040*	
H222	0.0938	0.0818	0.4233	0.045*	
H223	0.3338	−0.0020	0.4230	0.049*	
H224	0.5020	0.0702	0.3332	0.048*	
H225	0.4674	0.2287	0.1770	0.042*	
H226	0.5571	0.2250	0.2581	0.042*	
H231	0.1348	0.3830	0.4421	0.049*	
H232	0.2646	0.3868	0.5637	0.057*	
H233	0.5084	0.3941	0.5458	0.068*	
H234	0.6242	0.3831	0.4142	0.053*	
H235	0.5062	0.4466	0.2301	0.049*	
H236	0.6019	0.3476	0.2684	0.049*	

### Atomic displacement parameters (Å^2^)

**Table:**

	*U*^11^	*U*^22^	*U*^33^	*U*^12^	*U*^13^	*U*^23^
Ni1	0.0305 (4)	0.0184 (4)	0.0248 (4)	0.0000 (3)	−0.0049 (3)	−0.0102 (3)
Ni2	0.0351 (4)	0.0283 (4)	0.0278 (4)	0.0021 (3)	−0.0073 (3)	−0.0111 (3)
S12	0.0348 (7)	0.0291 (8)	0.0313 (8)	0.0000 (6)	−0.0100 (6)	−0.0072 (6)
S14	0.0492 (9)	0.0296 (8)	0.0433 (9)	−0.0088 (7)	0.0013 (7)	−0.0229 (7)
S22	0.0460 (9)	0.0325 (8)	0.0507 (10)	−0.0085 (7)	−0.0063 (7)	−0.0104 (7)
S24	0.0384 (8)	0.0407 (9)	0.0500 (10)	−0.0018 (7)	0.0032 (7)	−0.0218 (8)
O1	0.056 (3)	0.055 (3)	0.060 (3)	−0.010 (2)	−0.002 (2)	−0.013 (3)
N11	0.031 (2)	0.022 (2)	0.029 (2)	−0.002 (2)	−0.002 (2)	−0.010 (2)
N12	0.031 (2)	0.017 (2)	0.025 (2)	0.001 (2)	−0.007 (2)	−0.011 (2)
N13	0.030 (2)	0.018 (2)	0.027 (2)	−0.003 (2)	−0.004 (2)	−0.004 (2)
N14	0.031 (2)	0.020 (2)	0.030 (2)	0.002 (2)	−0.007 (2)	−0.015 (2)
N21	0.041 (3)	0.025 (3)	0.034 (3)	0.001 (2)	−0.009 (2)	−0.009 (2)
N22	0.033 (3)	0.032 (3)	0.027 (2)	0.003 (2)	−0.006 (2)	−0.017 (2)
N23	0.049 (3)	0.022 (2)	0.030 (3)	0.003 (2)	−0.012 (2)	−0.009 (2)
N24	0.034 (2)	0.028 (3)	0.037 (3)	−0.003 (2)	−0.004 (2)	−0.015 (2)
N122	0.037 (3)	0.023 (2)	0.027 (3)	0.000 (2)	−0.004 (2)	−0.011 (2)
N144	0.035 (2)	0.019 (2)	0.032 (3)	−0.001 (2)	−0.004 (2)	−0.010 (2)
N222	0.041 (3)	0.028 (3)	0.032 (3)	0.001 (2)	−0.006 (2)	−0.006 (2)
N244	0.039 (3)	0.030 (3)	0.037 (3)	0.003 (2)	−0.003 (2)	−0.008 (2)
C1	0.063 (4)	0.039 (4)	0.067 (5)	−0.011 (3)	−0.013 (4)	−0.003 (4)
C12	0.030 (3)	0.019 (3)	0.023 (3)	−0.006 (2)	0.003 (2)	−0.010 (2)
C14	0.026 (3)	0.023 (3)	0.030 (3)	−0.005 (2)	0.001 (2)	−0.006 (2)
C22	0.035 (3)	0.031 (3)	0.033 (3)	0.008 (3)	−0.009 (3)	−0.016 (3)
C24	0.041 (3)	0.034 (3)	0.025 (3)	−0.009 (3)	−0.006 (3)	−0.011 (3)
C111	0.043 (3)	0.024 (3)	0.031 (3)	−0.005 (3)	−0.004 (3)	−0.011 (3)
C112	0.040 (3)	0.027 (3)	0.044 (4)	−0.008 (3)	−0.011 (3)	−0.009 (3)
C113	0.038 (3)	0.024 (3)	0.040 (3)	0.000 (2)	−0.003 (3)	−0.004 (3)
C114	0.041 (3)	0.023 (3)	0.029 (3)	−0.001 (2)	0.002 (3)	−0.007 (2)
C115	0.037 (3)	0.025 (3)	0.023 (3)	0.000 (2)	−0.005 (2)	−0.009 (2)
C116	0.037 (3)	0.035 (3)	0.027 (3)	−0.007 (3)	−0.002 (2)	−0.016 (3)
C121	0.034 (3)	0.021 (3)	0.032 (3)	−0.005 (2)	−0.003 (2)	−0.015 (2)
C122	0.039 (3)	0.034 (3)	0.033 (3)	0.003 (3)	−0.019 (3)	−0.013 (3)
C123	0.047 (3)	0.021 (3)	0.042 (4)	0.004 (2)	−0.025 (3)	−0.008 (3)
C124	0.046 (3)	0.020 (3)	0.038 (3)	0.000 (2)	−0.009 (3)	−0.011 (3)
C125	0.026 (3)	0.026 (3)	0.029 (3)	−0.006 (2)	−0.003 (2)	−0.012 (2)
C126	0.043 (3)	0.021 (3)	0.027 (3)	0.004 (2)	−0.012 (2)	−0.013 (2)
C131	0.037 (3)	0.020 (3)	0.037 (3)	−0.002 (2)	−0.002 (3)	−0.008 (3)
C132	0.042 (3)	0.022 (3)	0.046 (4)	0.001 (3)	0.005 (3)	0.001 (3)
C133	0.037 (3)	0.023 (3)	0.054 (4)	−0.006 (2)	−0.004 (3)	−0.011 (3)
C134	0.036 (3)	0.023 (3)	0.050 (4)	−0.001 (2)	−0.014 (3)	−0.012 (3)
C135	0.032 (3)	0.018 (3)	0.031 (3)	−0.001 (2)	−0.007 (2)	−0.005 (2)
C136	0.032 (3)	0.027 (3)	0.036 (3)	−0.003 (2)	−0.007 (2)	−0.014 (3)
C211	0.043 (3)	0.031 (3)	0.045 (4)	−0.002 (3)	−0.009 (3)	−0.012 (3)
C212	0.056 (4)	0.041 (4)	0.040 (4)	−0.003 (3)	−0.021 (3)	−0.013 (3)
C213	0.054 (4)	0.039 (4)	0.033 (3)	0.002 (3)	−0.014 (3)	−0.018 (3)
C214	0.051 (4)	0.034 (3)	0.032 (3)	−0.005 (3)	0.004 (3)	−0.010 (3)
C215	0.043 (3)	0.026 (3)	0.033 (3)	−0.002 (3)	0.000 (3)	−0.013 (3)
C216	0.040 (3)	0.032 (3)	0.039 (4)	−0.006 (3)	0.000 (3)	−0.009 (3)
C221	0.031 (3)	0.036 (3)	0.036 (3)	−0.002 (3)	−0.004 (3)	−0.021 (3)
C222	0.044 (3)	0.032 (3)	0.043 (4)	−0.007 (3)	0.001 (3)	−0.020 (3)
C223	0.053 (4)	0.020 (3)	0.049 (4)	−0.004 (3)	−0.004 (3)	−0.010 (3)
C224	0.037 (3)	0.033 (3)	0.051 (4)	0.000 (3)	−0.009 (3)	−0.018 (3)
C225	0.038 (3)	0.027 (3)	0.039 (3)	−0.001 (3)	−0.014 (3)	−0.020 (3)
C226	0.030 (3)	0.031 (3)	0.039 (3)	0.001 (2)	−0.003 (3)	−0.010 (3)
C231	0.061 (4)	0.024 (3)	0.037 (4)	−0.005 (3)	−0.003 (3)	−0.011 (3)
C232	0.085 (5)	0.026 (3)	0.027 (3)	−0.003 (3)	−0.012 (3)	−0.006 (3)
C233	0.100 (6)	0.023 (3)	0.041 (4)	−0.010 (4)	−0.037 (4)	0.002 (3)
C234	0.060 (4)	0.030 (3)	0.040 (4)	−0.010 (3)	−0.020 (3)	−0.003 (3)
C235	0.047 (4)	0.025 (3)	0.034 (3)	−0.002 (3)	−0.018 (3)	−0.005 (3)
C236	0.035 (3)	0.038 (4)	0.054 (4)	−0.010 (3)	−0.010 (3)	−0.018 (3)

### Geometric parameters (Å, °)

**Table:**

Ni1—N11	2.079 (4)	C212—C213	1.362 (8)
Ni1—N12	2.086 (4)	C213—C214	1.389 (8)
Ni1—N13	2.093 (4)	C214—C215	1.383 (8)
Ni1—N14	2.122 (4)	C215—C216	1.518 (8)
Ni1—N122	2.073 (4)	C221—C222	1.360 (8)
Ni1—N144	2.040 (5)	C222—C223	1.373 (8)
Ni2—N21	2.049 (5)	C223—C224	1.399 (8)
Ni2—N22	2.079 (4)	C224—C225	1.384 (7)
Ni2—N23	2.061 (5)	C225—C226	1.498 (8)
Ni2—N24	2.126 (4)	C231—C232	1.398 (9)
Ni2—N222	2.102 (5)	C232—C233	1.380 (9)
Ni2—N244	2.043 (5)	C233—C234	1.391 (9)
S12—C12	1.640 (5)	C234—C235	1.391 (8)
S14—C14	1.636 (6)	C235—C236	1.512 (8)
S22—C22	1.661 (7)	O1—H1	1.002
S24—C24	1.635 (6)	C1—H11	0.950
O1—C1	1.416 (7)	C1—H12	0.950
N11—C111	1.345 (7)	C1—H13	0.950
N11—C115	1.348 (6)	C111—H111	0.950
N12—C121	1.349 (6)	C112—H112	0.950
N12—C125	1.341 (6)	C113—H113	0.950
N13—C131	1.338 (6)	C114—H114	0.950
N13—C135	1.339 (7)	C116—H115	0.950
N14—C116	1.483 (6)	C116—H116	0.950
N14—C126	1.491 (6)	C121—H121	0.950
N14—C136	1.481 (6)	C122—H122	0.950
N21—C211	1.340 (7)	C123—H123	0.950
N21—C215	1.355 (7)	C124—H124	0.950
N22—C221	1.350 (7)	C126—H125	0.950
N22—C225	1.353 (6)	C126—H126	0.950
N23—C231	1.351 (7)	C131—H131	0.950
N23—C235	1.350 (7)	C132—H132	0.950
N24—C216	1.483 (7)	C133—H133	0.950
N24—C226	1.502 (7)	C134—H134	0.950
N24—C236	1.479 (7)	C136—H135	0.950
N122—C12	1.153 (6)	C136—H136	0.950
N144—C14	1.155 (6)	C211—H211	0.950
N222—C22	1.165 (7)	C212—H212	0.950
N244—C24	1.160 (7)	C213—H213	0.950
C111—C112	1.381 (8)	C214—H214	0.950
C112—C113	1.382 (8)	C216—H215	0.950
C113—C114	1.391 (8)	C216—H216	0.950
C114—C115	1.379 (7)	C221—H221	0.950
C115—C116	1.522 (7)	C222—H222	0.950
C121—C122	1.381 (7)	C223—H223	0.950
C122—C123	1.387 (8)	C224—H224	0.950
C123—C124	1.368 (8)	C226—H225	0.950
C124—C125	1.389 (7)	C226—H226	0.950
C125—C126	1.505 (7)	C231—H231	0.950
C131—C132	1.374 (8)	C232—H232	0.950
C132—C133	1.388 (8)	C233—H233	0.950
C133—C134	1.381 (8)	C234—H234	0.950
C134—C135	1.385 (7)	C236—H235	0.950
C135—C136	1.509 (7)	C236—H236	0.950
C211—C212	1.381 (8)		
			
O1···C211^i^	3.211 (7)	O1···C226	3.428 (7)
O1···C216	3.260 (7)	O1···C212^i^	3.453 (8)
C132···C233	3.318 (8)	C1···C211^i^	3.461 (9)
C211···C212^ii^	3.364 (8)	C113···C134^v^	3.487 (7)
S22···O1^iii^	3.370 (5)	C211···C213^ii^	3.496 (8)
C1···C111^iv^	3.412 (8)		
			
N11—Ni1—N12	88.2 (2)	C221—C222—C223	119.0 (5)
N11—Ni1—N13	161.0 (2)	C222—C223—C224	119.1 (5)
N11—Ni1—N14	81.5 (2)	C223—C224—C225	119.3 (5)
N11—Ni1—N122	91.0 (2)	N22—C225—C224	120.6 (5)
N11—Ni1—N144	98.1 (2)	N22—C225—C226	117.8 (5)
N12—Ni1—N13	91.4 (2)	C224—C225—C226	121.6 (5)
N12—Ni1—N14	81.8 (2)	N24—C226—C225	114.3 (4)
N12—Ni1—N122	173.5 (2)	N23—C231—C232	121.8 (6)
N12—Ni1—N144	93.9 (2)	C231—C232—C233	117.4 (6)
N13—Ni1—N14	79.6 (2)	C232—C233—C234	121.1 (6)
N13—Ni1—N122	87.3 (2)	C233—C234—C235	118.5 (6)
N13—Ni1—N144	101.0 (2)	N23—C235—C234	120.9 (5)
N14—Ni1—N122	91.7 (2)	N23—C235—C236	117.3 (5)
N14—Ni1—N144	175.7 (2)	C234—C235—C236	121.7 (5)
N122—Ni1—N144	92.6 (2)	N24—C236—C235	111.4 (4)
N21—Ni2—N22	93.0 (2)	C1—O1—H1	101.59
N21—Ni2—N23	161.9 (2)	O1—C1—H11	109.49
N21—Ni2—N24	80.6 (2)	O1—C1—H12	109.47
N21—Ni2—N222	89.6 (2)	O1—C1—H13	109.47
N21—Ni2—N244	99.9 (2)	H11—C1—H12	109.47
N22—Ni2—N23	87.2 (2)	H11—C1—H13	109.47
N22—Ni2—N24	83.3 (2)	H12—C1—H13	109.45
N22—Ni2—N222	172.2 (2)	N11—C111—H111	119.12
N22—Ni2—N244	95.7 (2)	C112—C111—H111	119.11
N23—Ni2—N24	81.4 (2)	C111—C112—H112	120.11
N23—Ni2—N222	88.0 (2)	C113—C112—H112	120.11
N23—Ni2—N244	98.2 (2)	C112—C113—H113	120.84
N24—Ni2—N222	89.9 (2)	C114—C113—H113	120.84
N24—Ni2—N244	179.0 (2)	C113—C114—H114	120.37
N222—Ni2—N244	91.1 (2)	C115—C114—H114	120.36
Ni1—N11—C111	127.7 (4)	N14—C116—H115	108.96
Ni1—N11—C115	113.6 (3)	N14—C116—H116	108.95
C111—N11—C115	118.7 (4)	C115—C116—H115	108.97
Ni1—N12—C121	126.9 (3)	C115—C116—H116	108.96
Ni1—N12—C125	115.2 (3)	H115—C116—H116	109.46
C121—N12—C125	117.8 (4)	N12—C121—H121	118.44
Ni1—N13—C131	128.7 (4)	C122—C121—H121	118.44
Ni1—N13—C135	112.8 (3)	C121—C122—H122	120.99
C131—N13—C135	118.3 (4)	C123—C122—H122	120.99
Ni1—N14—C116	105.9 (3)	C122—C123—H123	120.21
Ni1—N14—C126	110.1 (3)	C124—C123—H123	120.20
Ni1—N14—C136	105.2 (3)	C123—C124—H124	120.42
C116—N14—C126	112.8 (4)	C125—C124—H124	120.42
C116—N14—C136	111.9 (4)	N14—C126—H125	108.21
C126—N14—C136	110.5 (4)	N14—C126—H126	108.21
Ni2—N21—C211	126.7 (4)	C125—C126—H125	108.22
Ni2—N21—C215	114.4 (4)	C125—C126—H126	108.22
C211—N21—C215	118.9 (5)	H125—C126—H126	109.46
Ni2—N22—C221	126.7 (3)	N13—C131—H131	118.50
Ni2—N22—C225	113.1 (3)	C132—C131—H131	118.51
C221—N22—C225	119.1 (5)	C131—C132—H132	120.63
Ni2—N23—C231	126.5 (4)	C133—C132—H132	120.63
Ni2—N23—C235	113.3 (4)	C132—C133—H133	120.68
C231—N23—C235	120.2 (5)	C134—C133—H133	120.68
Ni2—N24—C216	105.8 (3)	C133—C134—H134	120.43
Ni2—N24—C226	108.2 (3)	C135—C134—H134	120.43
Ni2—N24—C236	106.4 (3)	N14—C136—H135	109.32
C216—N24—C226	110.6 (4)	N14—C136—H136	109.32
C216—N24—C236	113.9 (4)	C135—C136—H135	109.32
C226—N24—C236	111.6 (4)	C135—C136—H136	109.32
Ni1—N122—C12	173.1 (4)	H135—C136—H136	109.46
Ni1—N144—C14	166.0 (4)	N21—C211—H211	118.99
Ni2—N222—C22	154.9 (4)	C212—C211—H211	118.99
Ni2—N244—C24	155.9 (4)	C211—C212—H212	120.50
S12—C12—N122	178.5 (4)	C213—C212—H212	120.50
S14—C14—N144	178.9 (5)	C212—C213—H213	119.91
S22—C22—N222	178.9 (5)	C214—C213—H213	119.91
S24—C24—N244	179.1 (5)	C213—C214—H214	120.91
N11—C111—C112	121.8 (5)	C215—C214—H214	120.91
C111—C112—C113	119.8 (5)	N24—C216—H215	109.15
C112—C113—C114	118.3 (5)	N24—C216—H216	109.14
C113—C114—C115	119.3 (5)	C215—C216—H215	109.14
N11—C115—C114	122.1 (5)	C215—C216—H216	109.14
N11—C115—C116	116.0 (4)	H215—C216—H216	109.47
C114—C115—C116	121.8 (5)	N22—C221—H221	118.60
N14—C116—C115	111.5 (4)	C222—C221—H221	118.61
N12—C121—C122	123.1 (5)	C221—C222—H222	120.49
C121—C122—C123	118.0 (5)	C223—C222—H222	120.48
C122—C123—C124	119.6 (5)	C222—C223—H223	120.45
C123—C124—C125	119.2 (5)	C224—C223—H223	120.45
N12—C125—C124	122.2 (5)	C223—C224—H224	120.32
N12—C125—C126	117.8 (4)	C225—C224—H224	120.33
C124—C125—C126	120.0 (5)	N24—C226—H225	108.26
N14—C126—C125	114.5 (4)	N24—C226—H226	108.25
N13—C131—C132	123.0 (5)	C225—C226—H225	108.25
C131—C132—C133	118.7 (5)	C225—C226—H226	108.25
C132—C133—C134	118.6 (5)	H225—C226—H226	109.46
C133—C134—C135	119.1 (5)	N23—C231—H231	119.07
N13—C135—C134	122.2 (5)	C232—C231—H231	119.08
N13—C135—C136	116.8 (4)	C231—C232—H232	121.29
C134—C135—C136	120.9 (5)	C233—C232—H232	121.28
N14—C136—C135	110.1 (4)	C232—C233—H233	119.48
N21—C211—C212	122.0 (5)	C234—C233—H233	119.46
C211—C212—C213	119.0 (5)	C233—C234—H234	120.75
C212—C213—C214	120.2 (5)	C235—C234—H234	120.75
C213—C214—C215	118.2 (5)	N24—C236—H235	108.99
N21—C215—C214	121.7 (5)	N24—C236—H236	108.99
N21—C215—C216	115.7 (5)	C235—C236—H235	108.99
C214—C215—C216	122.6 (5)	C235—C236—H236	108.98
N24—C216—C215	110.8 (4)	H235—C236—H236	109.46
N22—C221—C222	122.8 (5)		
			
Ni1—N11—C111—C112	−179.0 (4)	N24—Ni2—N21—C211	163.4 (5)
Ni1—N11—C115—C114	177.4 (4)	N24—Ni2—N21—C215	−17.8 (4)
Ni1—N11—C115—C116	−5.8 (5)	N24—Ni2—N22—C221	173.4 (4)
Ni1—N12—C121—C122	−178.8 (4)	N24—Ni2—N22—C225	5.7 (4)
Ni1—N12—C125—C124	179.3 (4)	N24—Ni2—N23—C231	−166.8 (5)
Ni1—N12—C125—C126	0.5 (5)	N24—Ni2—N23—C235	14.8 (4)
Ni1—N13—C131—C132	−171.9 (4)	N24—C216—C215—C214	−156.9 (5)
Ni1—N13—C135—C134	174.5 (4)	N24—C226—C225—C224	−161.4 (5)
Ni1—N13—C135—C136	−1.5 (5)	N24—C236—C235—C234	160.1 (5)
Ni1—N14—C116—C115	−36.1 (4)	N122—Ni1—N11—C111	−102.2 (4)
Ni1—N14—C126—C125	8.8 (5)	N122—Ni1—N11—C115	79.7 (3)
Ni1—N14—C136—C135	40.9 (4)	N122—Ni1—N12—C121	−178 (1)
Ni2—N21—C211—C212	176.9 (4)	N122—Ni1—N12—C125	2(2)
Ni2—N21—C215—C214	−176.5 (4)	N122—Ni1—N13—C131	101.8 (4)
Ni2—N21—C215—C216	1.0 (6)	N122—Ni1—N13—C135	−72.4 (3)
Ni2—N22—C221—C222	−164.7 (4)	N122—Ni1—N14—C116	−64.6 (3)
Ni2—N22—C225—C224	166.7 (4)	N122—Ni1—N14—C126	173.2 (3)
Ni2—N22—C225—C226	−16.5 (6)	N122—Ni1—N14—C136	54.1 (3)
Ni2—N23—C231—C232	−176.1 (4)	N144—Ni1—N11—C111	−9.4 (4)
Ni2—N23—C235—C234	176.3 (4)	N144—Ni1—N11—C115	172.5 (3)
Ni2—N23—C235—C236	0.7 (6)	N144—Ni1—N12—C121	3.1 (4)
Ni2—N24—C216—C215	−37.2 (5)	N144—Ni1—N12—C125	−176.8 (3)
Ni2—N24—C226—C225	−15.1 (5)	N144—Ni1—N13—C131	9.7 (4)
Ni2—N24—C236—C235	33.5 (5)	N144—Ni1—N13—C135	−164.5 (3)
N11—Ni1—N12—C121	−94.8 (4)	N144—Ni1—N14—C116	111 (2)
N11—Ni1—N12—C125	85.3 (3)	N144—Ni1—N14—C126	−12 (2)
N11—Ni1—N13—C131	−173.2 (4)	N144—Ni1—N14—C136	−131 (2)
N11—Ni1—N13—C135	12.6 (7)	N222—Ni2—N21—C211	−106.7 (4)
N11—Ni1—N14—C116	26.2 (3)	N222—Ni2—N21—C215	72.2 (4)
N11—Ni1—N14—C126	−96.1 (3)	N222—Ni2—N22—C221	144 (1)
N11—Ni1—N14—C136	144.8 (3)	N222—Ni2—N22—C225	−23 (1)
N11—C111—C112—C113	1.7 (8)	N222—Ni2—N23—C231	103.1 (4)
N11—C115—C114—C113	2.0 (8)	N222—Ni2—N23—C235	−75.4 (4)
N11—C115—C116—N14	29.4 (6)	N222—Ni2—N24—C216	−59.8 (3)
N12—Ni1—N11—C111	84.3 (4)	N222—Ni2—N24—C226	−178.3 (3)
N12—Ni1—N11—C115	−93.9 (3)	N222—Ni2—N24—C236	61.6 (3)
N12—Ni1—N13—C131	−84.6 (4)	N244—Ni2—N21—C211	−15.6 (5)
N12—Ni1—N13—C135	101.2 (3)	N244—Ni2—N21—C215	163.2 (4)
N12—Ni1—N14—C116	115.6 (3)	N244—Ni2—N22—C221	−6.3 (4)
N12—Ni1—N14—C126	−6.7 (3)	N244—Ni2—N22—C225	−173.9 (4)
N12—Ni1—N14—C136	−125.8 (3)	N244—Ni2—N23—C231	12.3 (5)
N12—C121—C122—C123	0.0 (8)	N244—Ni2—N23—C235	−166.2 (4)
N12—C125—C124—C123	−0.8 (8)	N244—Ni2—N24—C216	145 (10)
N12—C125—C126—N14	−6.5 (6)	N244—Ni2—N24—C226	27 (10)
N13—Ni1—N11—C111	173.4 (4)	N244—Ni2—N24—C236	−93 (10)
N13—Ni1—N11—C115	−4.7 (7)	C111—N11—C115—C114	−1.0 (7)
N13—Ni1—N12—C121	104.2 (4)	C111—N11—C115—C116	175.9 (4)
N13—Ni1—N12—C125	−75.7 (3)	C111—C112—C113—C114	−0.6 (8)
N13—Ni1—N14—C116	−151.5 (3)	C112—C111—N11—C115	−0.9 (7)
N13—Ni1—N14—C126	86.3 (3)	C112—C113—C114—C115	−1.2 (8)
N13—Ni1—N14—C136	−32.8 (3)	C113—C114—C115—C116	−174.6 (5)
N13—C131—C132—C133	−2.1 (8)	C115—C116—N14—C126	84.4 (5)
N13—C135—C134—C133	−1.1 (8)	C115—C116—N14—C136	−150.3 (4)
N13—C135—C136—N14	−27.7 (6)	C116—N14—C126—C125	−109.3 (5)
N14—Ni1—N11—C111	166.3 (4)	C116—N14—C136—C135	155.5 (4)
N14—Ni1—N11—C115	−11.9 (3)	C121—N12—C125—C124	−0.6 (7)
N14—Ni1—N12—C121	−176.5 (4)	C121—N12—C125—C126	−179.4 (4)
N14—Ni1—N12—C125	3.6 (3)	C121—C122—C123—C124	−1.4 (8)
N14—Ni1—N13—C131	−166.0 (4)	C122—C121—N12—C125	1.0 (7)
N14—Ni1—N13—C135	19.8 (3)	C122—C123—C124—C125	1.8 (8)
N14—C116—C115—C114	−153.8 (5)	C123—C124—C125—C126	178.0 (5)
N14—C126—C125—C124	174.7 (4)	C125—C126—N14—C136	124.6 (5)
N14—C136—C135—C134	156.3 (4)	C126—N14—C136—C135	−77.9 (5)
N21—Ni2—N22—C221	−106.5 (4)	C131—N13—C135—C134	−0.4 (7)
N21—Ni2—N22—C225	85.9 (4)	C131—N13—C135—C136	−176.4 (4)
N21—Ni2—N23—C231	−174.1 (5)	C131—C132—C133—C134	0.5 (8)
N21—Ni2—N23—C235	7.5 (8)	C132—C131—N13—C135	2.1 (7)
N21—Ni2—N24—C216	29.8 (3)	C132—C133—C134—C135	1.0 (8)
N21—Ni2—N24—C226	−88.7 (3)	C133—C134—C135—C136	174.7 (5)
N21—Ni2—N24—C236	151.3 (4)	C211—N21—C215—C214	2.4 (8)
N21—C211—C212—C213	1.6 (9)	C211—N21—C215—C216	179.9 (5)
N21—C215—C214—C213	−2.6 (8)	C211—C212—C213—C214	−1.8 (9)
N21—C215—C216—N24	25.6 (6)	C212—C211—N21—C215	−1.9 (8)
N22—Ni2—N21—C211	80.7 (5)	C212—C213—C214—C215	2.3 (9)
N22—Ni2—N21—C215	−100.5 (4)	C213—C214—C215—C216	−179.9 (5)
N22—Ni2—N23—C231	−83.1 (4)	C215—C216—N24—C226	79.7 (5)
N22—Ni2—N23—C235	98.5 (4)	C215—C216—N24—C236	−153.6 (4)
N22—Ni2—N24—C216	124.0 (3)	C216—N24—C226—C225	−130.5 (5)
N22—Ni2—N24—C226	5.5 (3)	C216—N24—C236—C235	149.6 (4)
N22—Ni2—N24—C236	−114.6 (3)	C221—N22—C225—C224	−1.9 (8)
N22—C221—C222—C223	−0.1 (9)	C221—N22—C225—C226	174.9 (5)
N22—C225—C224—C223	−0.5 (8)	C221—C222—C223—C224	−2.4 (9)
N22—C225—C226—N24	21.8 (7)	C222—C221—N22—C225	2.2 (8)
N23—Ni2—N21—C211	170.7 (5)	C222—C223—C224—C225	2.7 (9)
N23—Ni2—N21—C215	−10.4 (8)	C223—C224—C225—C226	−177.2 (5)
N23—Ni2—N22—C221	91.7 (4)	C225—C226—N24—C236	101.6 (5)
N23—Ni2—N22—C225	−76.0 (4)	C226—N24—C236—C235	−84.2 (5)
N23—Ni2—N24—C216	−147.9 (3)	C231—N23—C235—C234	−2.2 (8)
N23—Ni2—N24—C226	93.6 (3)	C231—N23—C235—C236	−177.8 (5)
N23—Ni2—N24—C236	−26.4 (3)	C231—C232—C233—C234	−3.2 (9)
N23—C231—C232—C233	0.5 (8)	C232—C231—N23—C235	2.2 (8)
N23—C235—C234—C233	−0.4 (8)	C232—C233—C234—C235	3.1 (9)
N23—C235—C236—N24	−24.3 (7)	C233—C234—C235—C236	175.0 (5)

Symmetry codes: (i) *x*+1, *y*, *z*; (ii) −*x*, −*y*+1, −*z*; (iii) −*x*+1, −*y*+1, −*z*; (iv) −*x*+1, −*y*, −*z*+1; (v) *x*−1, *y*, *z*.
